# Significance of Single-Nucleotide Variants in Long Intergenic Non-protein Coding RNAs

**DOI:** 10.3389/fcell.2020.00347

**Published:** 2020-05-25

**Authors:** Hecun Zou, Lan-Xiang Wu, Lihong Tan, Fei-Fei Shang, Hong-Hao Zhou

**Affiliations:** ^1^Institute of Life Sciences, Chongqing Medical University, Chongqing, China; ^2^Chongqing Medical and Pharmaceutical College, Chongqing, China; ^3^Xiangya Hospital, Central South University, Changsha, China

**Keywords:** single-nucleotide variant, long/large intergenic non-protein coding RNA, disease susceptibility, transcription, biological function

## Abstract

Single-nucleotide variants (SNVs) are the most common genetic variants and universally present in the human genome. Genome-wide association studies (GWASs) have identified a great number of disease or trait-associated variants, many of which are located in non-coding regions. Long intergenic non-protein coding RNAs (lincRNAs) are the major subtype of long non-coding RNAs; lincRNAs play crucial roles in various disorders and cellular models *via* multiple mechanisms. With rapid growth in the number of the identified lincRNAs and genetic variants, there is great demand for an investigation of SNVs in lincRNAs. Hence, in this article, we mainly summarize the significant role of SNVs within human lincRNA regions. Some pivotal variants may serve as risk factors for the development of various disorders, especially cancer. They may also act as important regulatory signatures involved in the modulation of lincRNAs in a tissue- or disorder-specific manner. An increasing number of researches indicate that lincRNA variants would potentially provide additional options for genetic testing and disease risk assessment in the personalized medicine era.

## Introduction

**FIGURE 1:**
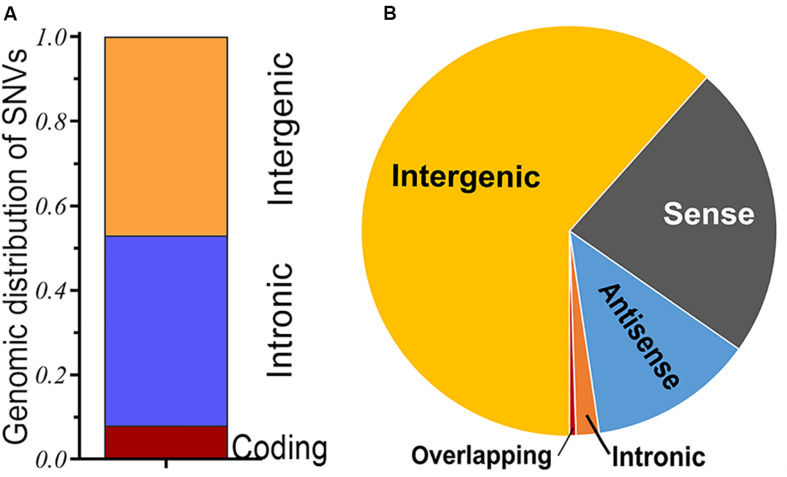
**(A)** Genomic distribution of single-nucleotide variants in cancers; a majority of cancer-associated single-nucleotide variants (SNVs) [single-nucleotide polymorphisms (SNPs)] are found in the intergenic or intronic regions, and only small numbers are located in protein-coding regions of the human genome. **(B)** Classification of long non-coding RNA (lncRNA) transcripts; long intergenic non-protein coding RNA (lincRNA) is a major subtype of lncRNA.

Single-nucleotide variant (SNV), also known as single-nucleotide polymorphism (SNP), is the variant of a single nucleotide that occurs at a specific genomic position. It is the most common type of genetic variants, which has long been confirmed in various loci of the genome (Human Genome Structural Variation Working Group, Eichler et al., 2007). In the past few decades, genetic variants have been typically used to dissect complex human disorders through research on candidate genes, particularly genome-wide association study (GWAS), an observational study of the genome-wide set of genetic variants in different individuals, which is performed to identify whether any variant is associated with the phenotypes. As a representative of a large-scale variant analysis, it has provided an approach to identifying potential genetic variant loci associated with heterogeneous disorders, including cancer susceptibility (Freedman et al., 2011). With the development of emerging technologies, such as microarray-based genotyping and high-throughput next-generation sequencing, it offers a novel avenue for the clinical application of genetic variants (GTEx Consortium et al., 2017). As might be expected, the role of genetic variants in understanding the pathogenesis of diseases, therapeutic response, and even ultimately personalized medicine will be indispensable in the near future. Based on the implementation of the International HapMap Project and the 1000 Genomes Project, great breakthroughs have been achieved in the research field of genetic variants, particularly focusing on some variants of protein-coding genes (Human Genome Structural Variation Working Group, Eichler et al., 2007; Genomes Project et al., 2015). However, genetic variants, especially SNVs, not only occur to protein-coding sequences, but many of them also fall within non-coding regions or the intergenic regions between two genes. For instance, a considerable genetic component has been confirmed to be involved in the susceptibility of various cancers; the genomic contexts of cancer-associated SNVs (SNPs) have been analyzed within a comprehensive GWAS catalog. Of these risk variants, less than 10% are mapped in protein-coding regions, whereas most of them are located in the intronic or intergenic regions (Figure 1A), it brings forward the issue of these non-coding loci and their importance role in cancer research (Hindorff et al., 2009).

The Human Genome Project (HGP) has determined the whole sequence of nucleotide base pairs that compose the human genome and initially provided approximately 20,000 proteins that could serve as therapeutic targets (Venter et al., 2001). Subsequent large-scale annotation efforts, such as the Encyclopedia of DNA Elements (ENCODE) project, surprisingly, have identified hundreds of thousands of non-coding RNAs, which were previously regarded as “junk DNA” (The Encode Project Consortium, 2012). Among them, a great quantity of long non-coding RNAs (lncRNAs) are transcribed in mammalian genomes. Based on their locations and characteristics, lncRNAs can be placed into five broad categories: (1) intergenic, (2) antisense, (3) sense, (4) intronic, and (5) overlapping (Ponting et al., 2009; Derrien et al., 2012). Thereinto, long/large intergenic non-protein coding RNAs (lincRNAs), which are located within the genomic interval between two coding genes, are the major subtypes of lncRNAs accounting for approximately 63% (Figure 1B). Compared with other lncRNAs, molecules for which we know next to nothing about, lincRNAs are generally unexplored and have yet to be elucidated. About half of these lincRNAs are transcribed from the vicinity (<10 kb) of protein-coding loci and more likely to be involved in *cis*-regulatory of the expression level of adjacent genes; other transcripts that are well away from an adjacent gene seem to have little chance of *cis*-regulatory within the nearby region. Although they rarely form triplexes within double-stranded DNA owing to their poor complementarity to sequences elsewhere within the genome, these lincRNAs often act as *trans*-regulatory players within some ribonucleoprotein complexes (Ulitsky and Bartel, 2013). LincRNAs may have crucial roles in various disorders and cellular models *via* multiple mechanisms. Alterations in the levels of lincRNA expression have been linked to the occurrence of various disorders, such as cancers; they may act as tumor suppressors or proto-oncogenes (Huarte, 2015). Currently, advances in high-throughput RNA sequencing and computing approaches allow for an unparalleled analysis of transcriptomes. Of the diverse kinds of RNA transcripts, lincRNAs are attractive as they can be found out from the existing RNA-seq datasets through available bioinformatics methods (Cabili et al., 2011).

According to recent reports from the ENCODE project, thousands and thousands of variant loci are present in the non-coding regions of the human genome, and total number continues to increase (Schaub et al., 2012). Generally, genetic variants, such as SNVs, which occur to the non-coding loci, are more frequently than in conservative protein-coding genes regions. A large number of GWAS-identified SNVs loci reside in the regions that encode lincRNAs, indicating that these variants of lincRNAs may play a crucial role in the susceptibility of diseases. More than three quarters of disease-associated genetic variants are remarkably overlapped in promoter or enhancer regions, suggesting that SNVs may serve as an important player in the regulation of transcript levels (Hindorff et al., 2009). Therefore, identification of such variant loci and elucidation of their biological functions would be of profound significance in understanding the etiology of disorders and in promoting novel approaches for the diagnosis, prevention, and treatment of disorder.

## Long Intergenic Non-Protein Coding RNA Variants and Disease Susceptibility

As a matter of fact, the occurrence of complex diseases (e.g., cancer) is related to multiple factors, including genetic, environmental, and lifestyle. Among them, genetic factors are of particular interest, just as GWASs and next-generation sequencing studies have greatly broadened the understanding of genetic variants that confer risk of diseases. Numerous genetic variants in lincRNA regions have been determined to be associated with the susceptibility of heterogeneous diseases, especially multiple types of cancer. Herein, we reviewed some lincRNAs that encompass disease or trait-associated variants (Tables 1, 2).

**TABLE 1:** Overviews of trait-associated variants on the chr8q24 locus.

LincRNA	Trait-associated variants	Diseases	Position	References
*CASC8*	rs378854	Adiposity	Intron	Ng et al., 2017
	rs10505477	Colorectal, gastric, and lung cancers	Intron	Ma et al., 2015; Hu et al., 2016
*CASC19*	rs138042437	Prostate cancer	Intron	Teerlink et al., 2016
*CCAT1*	rs6983267	Colorectal cancer, endometrial carcinoma	Enhancer	Kim et al., 2014; Zhao et al., 2016
*CCAT2*	rs6983267	Prostate, breast, colon, and colorectal cancers; myeloid malignancies	Exon	Yeager et al., 2007; Tuupanen et al., 2009; Ling et al., 2013; Shah et al., 2018
*PCAT1*	rs7463708	Prostate cancer	Enhancer	Guo et al., 2016
	rs10086908	Prostate cancer	Promoter	Guo et al., 2016
*PRNCR1*	rs1456315, rs7463708	Prostate cancer	Exon	Chung et al., 2011
	rs13252298, rs1456315	Colorectal cancer	Exon	Li et al., 2013
	rs183373024	Prostate cancer	Exon	Teerlink et al., 2016
*PVT1*	rs13281615	Breast cancer	Promoter	Zhang et al., 2014
	rs2720709, rs2648875	End-stage renal disease (ESRD)	Intron, intron	Hanson et al., 2007
	rs378854	Prostate cancer	Promoter	Meyer et al., 2011
	rs13255292, rs4733601	Diffuse large B cell lymphoma	Intron, downstream	Cerhan et al., 2014

*LincRNA, long intergenic non-protein coding RNA.*

**TABLE 2:** Overviews of other lincRNAs encompassing trait-associated variants.

LincRNA	Trait-associated variants	Diseases	Position	References
*CASC16*	rs3803662	Breast cancer, lung cancer	Exon	Orr et al., 2011
*CASC15*	rs6939340	Neuroblastoma	Intron	Maris et al., 2008
*GAS5*	rs145204276	Hepatocellular carcinoma (HCC), colorectal, and gastric cancers	Promoter	Tao et al., 2015; Li et al., 2018a
*H19*	rs217727	Coronary artery disease, type 2 diabetes	Exon	Gao et al., 2015
	rs2067051	Pneumoconiosis, coronary artery disease	Exon	Gao et al., 2015; Wu et al., 2016
	rs2107425	Ovarian and breast cancers, hypertrophic cardiomyopathy	Intron	Chu et al., 2016
	rs2839698	HCC, bladder, colorectal, and gastric cancer	Exon	Verhaegh et al., 2008; Chu et al., 2016; Yang et al., 2018
*HULC*	rs7763881, rs1041279	HCC	Intron	Wang et al., 2018a
*LINC00673*	rs11655237	Pancreatic cancer	Exon	Zheng et al., 2016
*LINC00951*	rs11752942	Esophageal squamous cell carcinoma (ESCC)	Exon	Wu et al., 2013
*LOC105378318*	rs1875147	Leprosy	Intron	Fava et al., 2017
*MALAT1*	rs619586	Pulmonary arterial hypertension (PAH), coronary atherosclerotic and congenital heart disease (CAD/CHD), breast cancer	Exon	Zhuo et al., 2017; Li et al., 2018b
	rs1194338	Colorectal cancer	Promoter	Li et al., 2017
	rs4102217	HCC	Promoter	Wang et al., 2018b
*MEG3*	rs941576, rs34552516	Type 1 diabetes (T1D)	Intron	Wallace et al., 2010; Westra et al., 2018
*MIAT*	rs2331291	Myocardial infarction	Intron	Ishii et al., 2006
	rs1894720	Paranoid schizophrenia	Exon	Rao et al., 2015
*PCGEM1*	rs6434568, rs16834898	Prostate cancer	Intron	Xue et al., 2013
*PCAT19*	rs11672691	Prostate cancer	Promoter	Gao et al., 2018
*PTCSC2*	rs965513	Papillary thyroid carcinoma (PTC)	Intron	He et al., 2015
*PTCSC3*	rs944289	PTC, large-vessel ischemic stroke	Promoter	Jendrzejewski et al., 2012; Lee et al., 2016
*TDRG1*	rs8506	ESCC, gastric cancer	Exon	Han et al., 2017
*TINCR*	rs2288947, rs8105637	Colorectal cancer, gastric cancer	Exon, intron	Zheng et al., 2017

*LincRNA, long intergenic non-protein coding RNA.*

### Long Intergenic Non-protein Coding RNA Variants on the chr8q24 Locus

Genome-wide association studies have pointed to the chr8q24 genomic locus as a hotspot for cancer-associated variants owing to the large density, more strength, and high allele frequency of these variants (Yeager et al., 2007; Tuupanen et al., 2009). Even though chromosome 8q24 has been considered as a “gene desert” region owing to the absence of functionally annotated genes, with the only notable exception of the frequently amplified *MYC* (a proto-oncogene involved in tumorigenesis) (Chung et al., 2011). Surprisingly, large-scale studies have revealed that several lincRNAs are transcribed from the chr8q24 locus, such as *CCAT1* (Kim et al., 2014), *CCAT2* (Ling et al., 2013), *PVT1* (Hanson et al., 2007), *PCAT1* (Guo et al., 2016), and *PRNCR1* (Li et al., 2013); all of these encompass multiple cancer-associated variants. For instance, lincRNA *CCAT2* (Colon Cancer-Associated Transcript 2, also termed *LINC00873*), a transcript spanning SNV rs6983267, is associated with an increased risk for prostate, breast, colon, and colorectal cancers (Yeager et al., 2007; Tuupanen et al., 2009; Ling et al., 2013). *CCAT2* is overexpressed in various types of cancers and may contribute to tumor growth, metastasis, and chromosomal instability by increasing MYC expression (Ling et al., 2013). LincRNA *PRNCR1* has been reported to be involved in prostate carcinogenesis and may play an oncogene role *via* modulating the androgen receptor (Chung et al., 2011), *PRNCR1* variants, especially rs1456315, are associated with the susceptibility of prostate and colorectal cancers (Li et al., 2013; Teerlink et al., 2016). Through an integrative analysis of the lncRNA transcriptome and GWAS data, Guo et al. (2016) have identified a prostate cancer-associated transcript *PCAT1* and 10 risk loci on the chr8q24.21, including *PCAT1* variants rs10086908 and rs7463708, which are significantly associated with prostate cancer susceptibility. As for *PVT1* (also termed *LINC00079*), a GWAS analysis has identified that its variants rs13255292 and rs4733601 are associated with the susceptibility of diffuse large B cell lymphoma (Cerhan et al., 2014). Other independent SNVs (e.g., rs2720709 and rs2648875), which are mapped on *PVT1*, especially contributes to the development of end-stage renal disease (ESRD) in patients with type 2 diabetes (Hanson et al., 2007). A recent meta-analysis has summarized the relationship between two common variants (rs10505477 and rs7837328) in the intronic region of *CASC8* (*LINC00860*) at 8q24 locus with the risk of cancers (Cui et al., 2018), including colorectal, gastric, and lung cancers (Ma et al., 2015; Hu et al., 2016). Another intronic loci rs378854 is related to adiposity in the individuals of African ancestry (Ng et al., 2017).

### Single-Nucleotide Variants in Long Intergenic Non-protein Coding RNA H19 Locus

The *H19* (also termed *LINC00008*) is located in chromosome 11p15.5, a paternally imprinted onco-fetal gene, which is typically down-regulated in adult tissues but can be overexpressed in multiple types of solid cancer. LincRNA *H19* expression is closely related to tumor growth, metastasis, recurrence, and clinical prognosis (Ge et al., 2018). *H19* variants are involved in the susceptibility of multiple diseases. A meta-analysis study has indicated that variant T allele of rs2107425 is correlated with a decreased risk of developing cancers (e.g., breast, ovarian, lung, and bladder cancers) (Chu et al., 2016; Wu et al., 2017), whereas variant rs2839698 is associated with an increased risk of digestive cancers (colorectal and gastric cancers) *via* up-regulating *H19* expression; of note, there is no significant association observed between rs217727 variant and cancers susceptibility (Chu et al., 2016). However, in other reports, *H19* rs217727 has been linked to the risk of hepatocellular carcinoma (HCC) (Ge et al., 2018), oral squamous cell carcinoma (OSCC), and bladder cancer in the Chinese population (Guo Q. Y. et al., 2017). For coronary artery disease (CAD), the T variant of rs217727 is associated with an increased risk, whereas rs2067051 A variant is linked to a decreased risk (Gao et al., 2015). *H19* rs217727, but not rs2107425 variant, is associated with susceptibility of women with preeclampsia (PE) (Harati-Sadegh et al., 2018). Additionally, maternally transmitted fetal *H19* variants (e.g., rs217727, rs2071094, and rs10732516), along with paternal *IGF2* variants, are independently correlated with the placental DNA methylation levels (Marjonen et al., 2018) and birth weight of newborns (Petry et al., 2011).

### Single-Nucleotide Variant in *MALAT1* and *MIAT* Regions

LincRNA *MALAT1* (metastasis-associated lung adenocarcinoma transcript 1, also termed *LINC00047*) has rs619586 A > G variant, which is significantly associated with the susceptibility of pulmonary arterial hypertension (PAH), and the carriers with variant G genotypes have a decreased PAH risk (Zhuo et al., 2017). Recent study has suggested that rs619586 AG/GG genotypes could reduce the risks of coronary atherosclerotic heart disease and congenital heart disease (CHD) by regulating *MALAT1* expression (Li et al., 2018b). Another report has showed that *MALAT1* is overexpressed in colorectal cancers and that SNV rs1194338 mapping to its promoter region is significantly associated with a decreased risk of colorectal cancer (Li et al., 2017). Moreover, the large-scale case–control association studies have identified a novel myocardial infarction-associated transcript, *MIAT* (also termed *LINC00066*), which encompasses rs2331291, and other variants confer the susceptibility of myocardial infarction (Ishii et al., 2006). As a component of the nuclear matrix, *MIAT* is mainly expressed in neurons, Rao et al. (2015) have reported that SNV rs1894720 is correlated with paranoid schizophrenia susceptibility, and MIAT may contribute to the pathogenesis of schizophrenia.

### Other Long Intergenic Non-protein Coding RNA Variants in Human Cancers

In addition to the above lincRNA molecules, recent studies have identified many other cancer-associated variants within lincRNA regions. For example, the tissue differentiation-inducing non-protein coding RNA (*TINCR*), also termed *LINC00036*, is essential for somatic tissue differentiation and tumor progression (Kretz et al., 2013). It has been demonstrated that two variants of *TINCR* (rs2288947 and rs8105637) are significantly correlated with the susceptibility and lymph node metastasis of colorectal cancer (Zheng et al., 2017); the lincRNA *TINCR* rs2288947 G allele and rs8113645 A allele genotypes could reduce the risk of gastric cancer. *HULC*, an HCC up-regulated lncRNA, also termed *LINC00078*, and its variants (rs7763881 and rs1041279) are linked to the susceptibility of HCC (Wang et al., 2018a). In thyroid carcinoma, several papillary thyroid carcinoma susceptibility candidates, such as *PTCSC2*, contain a risk-variant rs965513, and *PTCSC3* encompasses rs944289; two lincRNA expression levels are strongly down-regulated in thyroid carcinoma tissues (Jendrzejewski et al., 2012; He et al., 2015). Additionally, GWAS analyses have identified five tag-SNVs, including rs944289 located in *PTCSC3*, are associated with large-vessel ischemic stroke (Lee et al., 2016). Xue et al. (2013) have reported that a prostate cancer gene expression marker, *PCGEM1* (*LINC00071*), containing two risk-SNVs (rs6434568 C and rs16834898 A alleles) that are associated with a decreased risk of prostate cancer. Another prostate cancer risk-associated allele rs75823044 mapping to promoter of *LINC00676* is almost exclusively found in African ancestry populations (Conti et al., 2017). In a GWAS analysis, five common variants including rs3803662 on the exon of *CASC16* (*LINC00918*) have been identified to contribute to the susceptibility of lung and breast cancers (Orr et al., 2011). Furthermore, the colorectal cancer risk-SNV rs11776042 is located in the promoter of *LNC00964*, in which lincRNA is significantly decreased in colorectal cancer tissues (Chu et al., 2015). For tumor suppressor lncRNA *GAS5*, an insertion/deletion variant of rs145204276 is associated with the susceptibility of HCC (Tao et al., 2015) and colorectal and gastric cancers (Li et al., 2018a).

### Other Disease-Associated Variants in Long Intergenic Non-protein Coding RNA Regions

Except for cancer susceptibility, some lincRNA variants are found to be associated with the risk of other heterogeneous diseases. GWAS and expression quantitative trait locus (eQTL) analyses have identified a risk factor for pathological inflammatory responses of leprosy, SNV rs1875147, which is an eQTL variant for lincRNA *LOC105378318* located in chromosome 10p21.2 (Fava et al., 2017). Rautanen et al. have found a variant rs140817150 in the intron of *LOC107986770*, which may be correlated with bacteremia susceptibility in African children (Kenyan Bacteraemia Study Group et al., 2016). A systematic analysis highlights some variant loci in lncRNA regions linked to cardiometabolic disorders; one of them, lincRNA *LOC157273* harboring rs4841132, is linked to the regulation of serum lipid cholesterol (Ghanbari et al., 2018). Shyn et al.’s (2011) GWAS analysis has identified a major depressive disorder (MDD) risk-associated variant rs12526133, which resides in exon of *LINC01108*, in which lincRNA is overexpressed in patients with MDD. Moreover, the maternally expressed imprinted gene, *MEG3* (also termed *LINC00023*), containing variants rs941576 (Wallace et al., 2010) and rs34552516 (Westra et al., 2018), which is found to be associated with susceptibility of type 1 diabetes. Nikpay et al.’s (2015) comprehensive GWAS meta-analyses have reported an association of CAD susceptibility with several SNVs, such as rs1870634, which is located in the downstream of *LINC00841*, and its GG genotype is strongly linked to CAD risk and has a higher frequency in CAD patients.

## Long Intergenic Non-Protein Coding RNA Variants and Clinicopathological Characteristics, Prognosis, and Treatment Response

### For Clinicopathological Characteristics and Prognosis

In addition to disease susceptibility, trait-associated SNVs are widely used for the indication of clinicopathological characteristics, prognosis, and treatment response (Gong et al., 2017). For example, with regard to a neuroblastoma-associated variant rs6939340, which is mapped on the intronic locus of lincRNA *CASC15* and *NBAT1*, neuroblastoma individuals with the risk alleles are more likely to have clinical aggressive presentation, including metastatic disease, tumor with *MYCN* amplification, and disease relapse (Maris et al., 2008). Two independent cohort studies have observed that risk-SNV rs2608053 of *PVT1* is correlated to the survival outcome of patients with classical Hodgkin lymphoma (Ghesquieres et al., 2018). For multiple sclerosis, several risk loci of *PVT1* may contribute to the prediction of an optimal response to treatment with glatiramer acetate (Kulakova et al., 2017). LincRNA *H19* variants have been found to increase the risk of ischemic strokes, and the up-regulated *H19* may induce cerebral ischemia reperfusion injury by activating autophagy (Wang et al., 2017). Recent studies have reported that *H19* rs2839698 variant may serve as an indicator for the increased risk and poor prognosis of HCC (Yang et al., 2018). Among individuals with coal workers’ pneumoconiosis (CWP), carriers of *H19* rs2067051 CT/TT genotypes are associated with a decreased risk; *H19* rs2067051 may be a possible biomarker for CWP prevention (Wu et al., 2016). A case–control study has shown that lincRNA *MALAT1* variant rs4102217 is related to increased HCC risks; this SNV may be a potential predictor for the risk and prognosis of patients with HCC (Wang et al., 2018b). Another *MALAT1* rs3200401 T allele has been found to confer better survival for patients with advanced lung adenocarcinoma (Wang et al., 2017). Furthermore, *TDRG1* (testis development related 1, also termed *LINC00532*) is overexpressed in esophageal squamous cell carcinoma (ESCC) tissues; the AA genotype of variant rs8506 is linked to an increased risk of ESCC; this risk allele may regulate TDRG1 expression by disrupting the sponge binding of miR-526b; high TDRG1 expression and rs8506 A allele variant may contribute to the advanced tumor–node–metastasis stage and poor survival for ESCC patients (Han et al., 2017). Recent GWAS analyses have demonstrated that variant rs11672691 of *PCAT19* (*LINC01190*) on 19q13 is positively related to aggressive prostate cancer. Further cohort studies have confirmed the association of rs11672691 with clinical characteristics of aggressive disease, including high tumor stage, prostate-specific antigen (PSA) progression, and development of castration-resistant prostate cancer (CRPC). The risk GG genotype of rs11672691 is also associated with a poor prognosis for patients with prostate cancer (Gao et al., 2018). These results highlight the clinical potential of trait-associated SNV, which may serve as risk stratification markers for the management of cancer patients.

### Indication of Treatment Response

Recent GWAS analyses have identified two common SNVs (rs4476990 and rs3802201), in which mapping to *MIR2052HG* may affect the recurrence risk of breast cancer patients treated with aromatase inhibitors. Expressions of *MIR2052HG* and estrogen receptor α (ERα, encoded by *ESR1* gene) are induced by aromatase inhibitors and estrogen in a variant-dependent manner. *MIR2052HG* could sustain the levels of ERα *via* promoting AKT/FOXO3-mediated *ESR1* transcription and limiting the ubiquitin-mediated ERα degradation. Its risk variant genotypes could enhance ERα binding to estrogen response elements and result in an alteration of response to aromatase inhibitors treatment for cancer patients (Ingle et al., 2016). In the evaluation of adverse reaction for lung cancer patients receiving platinum-based chemotherapy, the variants *CASC8* rs10505477 (Hu et al., 2016) and *ANRIL* rs1333049 are correlated with overall toxicity, especially severe hematologic and gastrointestinal toxicity; lincRNA *MEG3* rs116907618 is correlated with severe gastrointestinal toxicity; these variants may be considered as biomarkers for the evaluation of platinum-based treatment (Gong et al., 2017). Moreover, the rs10505477 GG genotype of *CASC8* is also associated with tumor size, lymph node metastasis, and tumor–node–metastasis stage and may contribute to the survival for gastric cancers patients (Ma et al., 2015). In nasopharyngeal carcinoma (NPC), lncRNA *GAS5* variant rs2067079 is associated with an increased risk of severe myelosuppression and neutropenia, whereas rs6790 may decrease the incidence rate of toxic reactions induced by chemo-radiotherapy in NPC patients (Guo Z. et al., 2017). Functional genomic studies have revealed that *GAS5* promoter encompassing SNV rs55829688 (T > C), which up-regulates *GAS5* expression *via* interacting with transcription factor TP63, may aggravate myelosuppression and result in a poor prognosis for patients with acute myeloid leukemia (AML) (Yan et al., 2017). Additionally, GWAS analyses have identified that some genetic variants are correlated with the pharmacokinetics of psychotropic drugs, such as variant rs16935279 located in an intron of LINC01592; its C allele carriers have a lower metabolism rate for anti-epileptic drugs (Athanasiu et al., 2015).

## Long Intergenic Non-Protein Coding RNA Variants Regulate Gene Transcription

**FIGURE 2:**
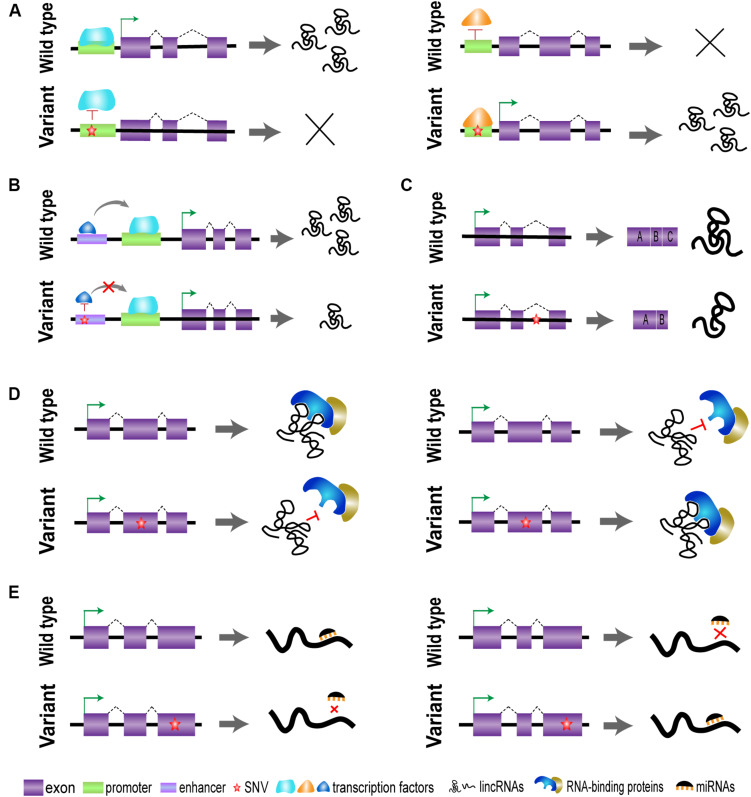
Graphical representations of the driving effect of variants [single-nucleotide variants (SNVs)] on long intergenic non-protein coding RNA (lincRNA) regions. **(A)** Genetic variants located in promoter may affect the binding of transcription factors and regulate the transcription of lincRNAs. **(B)** Genetic variants on enhancer affect the binding of transcription co-regulators and regulate lincRNA expression. **(C)** Genetic variants on intron may affect the process of splicing and stability of lincRNA conformation. **(D)** Genetic variants located in exons affect the lincRNA secondary structure, lincRNA stability, and interactive function. **(E)** Genetic variants on exons may affect the sponging of microRNAs (miRNAs).

Genome-wide association studies have identified a lot of trait-associated variants, most of which reside in non-coding regions of the human genome. However, the specific functional mechanism of genetic variants still remains confused, which is one of the major challenges for post-GWAS research (Schaub et al., 2012). The regulatory elements are mainly located within regions of non-coding DNA and play critical roles in the transcription of target genes. Emerging studies have showed that these regulatory elements can affect the expression of lincRNAs and other related genes *via* long-range chromatin interactions in a cell-type- or tissue-specific manner. Many genetic variants reside in the regulatory element regions of lincRNAs and may disrupt the interaction of transcription factors with a region containing SNVs (Figures 2A,B). The mapping of SNVs to lincRNA regulatory regions (especially promoters and enhancers) may indicate a potential impact of these variants on the transcription of target genes (GTEx Consortium et al., 2017).

### Single-Nucleotide Variants in Super-Enhancer Locus of *MYC* Gene

Many genetic variants are located in the upstream of *MYC*, a gene desert on 8q24, which is related to the susceptibility of multiple cancers. Some observations, such as chromosome conformation capture (3C) assays, histone acetylation, and methylation marks analyses, have demonstrated that these regulatory regions containing SNVs may serve as enhancers for *MYC* gene in a tissue-specific manner. Functional investigations suggest that lincRNA *CCAT2* augments the binding of transcription factor (TCF7L2 or TCF4) to *MYC* promoter region, activates WNT signaling, and increases the expression of target genes, especially the *MYC* proto-oncogene (Pomerantz et al., 2009; Ling et al., 2013). Although there is a disputable association between variant rs6983267 and *MYC* expression (Tuupanen et al., 2009), its risk G alleles produce more *CCAT2* transcripts, which are exclusively retained in the nucleus. Interestingly, a risk-SNV rs6983267 also contributes to increased expression of *CCAT1* (Zhao et al., 2016); an adjacent lincRNA of *CCAT2*, through affecting the long-range chromosomal interaction of *MYC* enhancer or *CCAT1* promoter, then results in a cell-cycle regulation and tumor development (Kim et al., 2014). Guo et al. (2016) have reported that a prostate cancer risk-associated T allele of rs7463708 at lincRNA *PCAT1* exhibited enhancer activity, through modulating the binding of novel transcription factor ONECUT2 with a distal enhancer that loops to the *PCAT1* promoter; this process increases *PCAT1* expression upon prolonged androgen treatment and promotes prostate transformation. Moreover, another prostate cancer risk-SNV rs378854 G alleles are also found to increase the expression of *PVT1* oncogene by regulating an interaction of transcription factor YY1 with the promoters of *PVT1* or *MYC* genes (Meyer et al., 2011). Similarly, the GG genotypes of rs13281615 increase *PVT1* transcription and promote cell proliferation in breast cancer (Zhang et al., 2014). Overexpression of *PVT1* may contribute to high levels of MYC mRNA and protein, along with an increased copy number, eventually leading to tumorigenesis (Zou et al., 2017). These results demonstrate the association of genetic variants with lincRNA transcription, although further studies are needed to reveal the relationship of these SNVs and lincRNA*s* on chromosome 8q24 locus.

### Single-Nucleotide Variants in Promoter Regions

Some SNVs reside in gene promoter regions and may influence the transcriptional expression of their target genes. Through an eQTL analysis of candidate genes and genetic variants in different tissues, an endometriosis risk-SNV rs3820282 is found to down-regulate *LINC00339* expression by affecting the activity of *LINC00339* promoter (Powell et al., 2016). Tao et al. (2015) have reported that an indel variant rs145204276 in the promoter region of *GAS5* contributes to the up-regulation of *GAS5 via* affecting the methylation status of *GAS5* promoter and regulating its transcriptional activity, thereby bringing its proto-oncogene role into play. Furthermore, the variant rs944289 of *PTCSC3* is reported to reside in a binding site for CCAAT/enhancer binding proteins (C/EBPα/β); this variant may affect the activity of *PTCSC3* promoter and down-regulate its transcript, then resulting in an abnormal expression of downstream genes and the progression of papillary thyroid carcinoma (Jendrzejewski et al., 2012).

Notably, a gene promoter region is likely to overlap with another super-enhancer locus, suggesting it that may have enhancer-like roles. In these interactions, lincRNA loci may serve as both target genes of its SNVs and the distal regulatory elements of other related genes. Integrative functional genomic and epigenomic analyses have identified that osteoporosis risk-associated SNV rs6426749 may act as a distal variant-specific enhancer and play a pivotal role in bone metabolism. Risk rs6426749 G alleles can affect the enhancer activity by binding to transcription factor TFAP2A; a thin process may increase transcription of *LINC00339* and modulate the expression of downstream gene *via* long-range chromatin loop formation in osteoblast cells (Chen et al., 2018). Recent studies have reported that prostate cancer risk-associated G allele of rs11672691 is associated with an increased expression of lincRNA *PCAT19* and oncogene CEACAM21; SNV rs11672691 is located in an enhancer element and may alter the binding site of its oncogenic transcription factor HOXA2. CRISPR/Cas9-mediated interference and activation assays have demonstrated that rs11672691 variant is involved in the regulation of its eQTL genes *PCAT19* and CEACAM21 expression and affects the cells’ aggressive property in prostate cancers (Gao et al., 2018). In another alternative mechanism, risk variant rs11672691 is associated with the decreased levels of a short isoform of *PCAT19* (*PCAT19*-short) and increased levels of a long isoform (*PCAT19*-long). This risk SNV locus is bifunctional with both promoter and enhancer activity, which maps to a promoter of *PCAT19*-short and the third intron of *PCAT19*-long. Risk allele rs11672691 and its linkage disequilibrium SNV rs887391 may alter the binding profiles of transcription factors NKX3.1 and YY1, thereby elevating the abundance of *PCAT19*-long through promoter-enhancer switching. Ultimately, it gives rise to an increased formation of the HNRNPAB-*PCAT19*-long complex to activate a subset of cell-cycle genes and promote prostate cancer aggression (Hua et al., 2018).

Another causative *cis*-regulatory mechanism has been constructed *via* integrative genomic analyses; the breast cancer-associated variant rs4415084 is located in a GATA3-binding motif of *LINC02224*, which refers to the differential GATA3 binding and chromatin accessibility, thereby promoting the transcription of *LINC02224* and *MRPS30* genes (Zhang et al., 2018). It is reasonable to postulate that the interactions of lincRNA, trait-associated variants, and regulatory factor may contribute to the development of specific disorders.

## Single-Nucleotide Variants Affect the Biological Function of Long Intergenic Non-Protein Coding RNA

Currently, genetic variants in potential lincRNA regions have attracted increasing interest; it has been established that many SNVs are associated with susceptibility of multiple diseases. It is evident that the expression and function of lincRNAs may be influenced by its SNVs in a cell-type- or tissue-specific manner. A comprehensive analysis has suggested that genetic variants in lincRNA regions also possibly affect the process of splicing and stability of lincRNA conformation, thereby leading to a modification of their interacting partners, as shown in Figures 2C–E (Hon et al., 2017).

### Effect of Single-Nucleotide Variants on the Role of Long Intergenic Non-protein Coding RNA CCAT2

Several observations, such as eQTL and DNAase peak assays, indicate that genetic variants that occurred in exons of lincRNAs may change the lincRNA secondary structure, thereby affecting its stability, interactive properties, and regulatory functions (Khurana et al., 2016). For example, lincRNA *CCAT2* could act as a scaffold or assembly platform and modulate the alternative splicing of glutaminase (GLS) pre-mRNA *via* directly binding to a Cleavage Factor I (CFIm) complex. However, SNV rs6983267 (G/T) may affect the interaction of CCAT2 with CFIm complex by changing lincRNA secondary structure and initiating a domino effect mechanism; this process leads to allele-specific reprogramming of cellular energy metabolism in colon cancers (Redis et al., 2016). Moreover, by using allele-specific *CCAT2* transgenic mice, recently, Shah et al. (2018) have revealed that overexpression of *CCAT2* may lead to genomic instability and myeloid malignancies; the SNV rs6983267-specific RNA-editing induces the dysregulation of a genome-wide gene expression by down-regulating EZH2, a histone-lysine *N*-methyltransferase, which then results in the impairment of immune processes and development of myelodysplastic neoplasms *in vivo*. In another study, Sur IK and his colleagues have generated mice lacking a *myc* enhancer region spanning risk-SNV rs6983267; the mutant mice have not showed an overt phenotype but confer resistance to intestinal tumorigenesis induced by APC^*min mutation*^ (Sur et al., 2012). These studies indicate that cancer risk-associated variants identified from the human genome may also exert a functional effect for animals *in vivo*.

### Effect of Single-Nucleotide Variants on the Long Intergenic Non-protein Coding RNA Secondary Structure

It is worth noting that lincRNAs have a long average length and that their exon regions contain numerous trait-associated variants; significant alterations of lincRNA secondary structure may be caused by its SNVs on exon loci. Many variants such as *PRNCR1* (prostate cancer-associated non-coding RNA) are located in exon regions, for example, rs1456315 G/A; it has been predicted to affect the lincRNA secondary structure of *PRNCR1* (Chung et al., 2011) and then alter lincRNA stability and conformation, even giving rise to the modification of its interacting partners. Xue et al. (2015) have also reported that SNV rs7958904 G/C in an exon region does not affect transcription activity of *HOTAIR*; however, in *in silico* analyses, it is shown to alter the RNA secondary structure of *HOTAIR*. These findings indicate that genetic variants, especially SNVs in exon loci, may play a different role *via* affecting the lincRNA structure.

### Effect of Single-Nucleotide Variants on MicroRNA Binding

Not surprisingly, it has been documented that some microRNAs (miRNAs) can function in a non-canonical manner to regulate lincRNA expression levels or directly interact with lincRNA molecules. The competing endogenous RNA (ceRNA) is a mechanism that lncRNA could competitively bind or sponge miRNAs, such as ceRNA *MALAT1*; its exon locus contains a variant rs619586 A > G, which can significantly up-regulate the expression of XBP1 (X box-binding protein 1) by sponging miR-214 and then suppressing the proliferation and migration of vascular endothelial cells *in vitro* (Zhuo et al., 2017). In another case, variant rs11752942 of *LINC00951* exon is linked to the susceptibility of ESCC; risk G alleles of rs11752942 may decrease the expression levels of *LINC00951 via* affecting the binding of miR-149-3p, thereby regulating cell proliferation and tumor growth (Wu et al., 2013). Intriguingly, recent studies have demonstrated that pancreatic cancer risk-SNV rs11655237 G > A in the *LINC00673* exon region is likely to create a target site for miR-1231 binding and reduces the function of LINC00673 in an allele-specific manner. Down-regulation of LINC00673 may attenuate the interaction of PTPN11 with an E3 ubiquitin ligase PRPF19 and suppress the ubiquitin-mediated PTPN11 degradation; these processes enhance an oncogenic signaling whereas diminish STAT1-dependent anti-oncogenic signaling in cancer cells (Zheng et al., 2016). These findings highlight the regulatory relationships of miRNAs with lincRNAs in a variant-specific manner and may offer a wider field for future research on lincRNA.

## Approaches for Identifying Drivers

As summarized above, genetic variants play a very significant role in the transcription and biological function of lincRNAs, contributing to various disease susceptibility, progression, prognosis, and treatment response. Genetic variants may act as a driving factor to affect the role of lincRNAs; just like a driver who drives a vehicle, analogously, lincRNA variants may vividly serve as a putative driver to regulate the lincRNA molecules.

### Computational Approaches

Driver identification is a challenging task, owing to their complex and diverse modes of action and the inadequate understanding of non-coding regions; the computational prediction of non-coding drivers is even more challenging than that of protein-coding drivers. In addition, non-coding variants are more abundant than protein-coding genes; hence, the key variants with functional significance have to be distinguished from a larger set of passenger events (Khurana et al., 2016). Currently, several online databases have been constructed to describe genomic variants in lncRNA regions, such as lincSNP, lncRNASNP2, and LncVar. More specifically, lincSNP 2.0 is an integrated database to identify and annotate disease-associated SNVs on human lincRNAs and their transcription factor binding sites (Ning et al., 2017). LncRNASNP2 is an updated database of comprehensive information about SNVs or mutations in human and mouse lncRNAs, as well as their impacts on lncRNA structure and potential function on miRNA binding (Miao et al., 2018). LncVar provides genetic variants associated with lncRNAs in multiple species and their effects on biological function of lncRNAs (Chen et al., 2017). Furthermore, a large number of GWAS analyses have successfully identified an array of genetic variants that are associated with various types of human disorders (MacArthur et al., 2017). Numerous public databases have been set out to provide a comprehensive description of genetic variants and GWAS data in the human genome with high impact (Genomes Project et al., 2015). A brief overview of these databases with their key features and corresponding references is presented in Table 3.

**TABLE 3:** Some databases that may be used for driver identification.

Tools	Functional annotation	Link	References
LincSNP 2.0	Store and annotate disease-associated SNVs in human lncRNAs and their transcription factor binding sites (TFBSs)	http://bioinfo.hrbmu.edu.cn/LincSNP	Ning et al., 2017
lncRNASNP2	Comprehensive information of SNVs and mutations in lncRNAs, as well as their impacts on lncRNA structure and function	http://bioinfo.life.hust.edu.cn/lncRNASNP2	Miao et al., 2018
LncVar	A database of genetic variation associated with long non-coding genes in six species	http://bioinfo.ibp.ac.cn/LncVar	Chen et al., 2017
The 1000 Genomes Project	The largest public catalog of human variation and genotype data	http://www.internationalgenome.org/	Genomes Project et al., 2015
dbSNP	A public-domain archive for a broad collection of simple genetic polymorphisms	https://www.ncbi.nlm.nih.gov/snp	
GWAS Catalog	A catalog that has provided data from published genome-wide association studies	www.ebi.ac.uk/gwas/	MacArthur et al., 2017
GWAS4D	A web server that systematically evaluates GWAS signals and identifies context-specific regulatory variants	http://mulinlab.tmu.edu.cn/gwas4d	Huang et al., 2018

*SNVs, single-nucleotide variants; lncRNAs, long non-coding RNAs; GWAS, genome-wide association study.*

Functional annotations and linkage disequilibrium analyses of genetic variants can be performed based on public databases and bioinformatic methods. Among tag-SNVs with strong linkage disequilibrium, significant genotype-specific effects on lincRNA expression can be observed by eQTL analysis (GTEx Consortium et al., 2017). Subsequently, according to ChIP-Seq data from the ENCODE database^1^, some trait-associated SNVs can be picked out; those variants mapping to *cis*-regulatory motifs may affect the binding activities of many interrelated transcription factors, including EZH2, CHD1, TCF7L2, and CTCF. These transcription factors may be closely related to the occurrence and progression of various human disorders, such as enhancer of zeste homologue 2 (EZH2), which is overexpressed in several human tumors and accounts for the aggressiveness and unfavorable prognoses of various cancers.

### Function Verification

Many functional verification studies of genetic variants have focused on protein-coding regions of the human genome. With an expanding appreciation that non-coding variants play a crucial role in the development of disorders, several recent studies have set out to explore approaches to evaluate the function of non-coding variants (Khurana et al., 2016). For example, experimental methods used to understand the effects of *cis*-regulatory variants within a promoter or enhancer region on cellular biological functions is summarized as follows. A main strategy is required to introduce the sequence variants, the mutated DNA fragment can be constructed *via* site-directed mutagenesis, CRISPR–Cas system (Konermann et al., 2015) or oligonucleotide synthesis. Subsequently, the functional output of non-coding variants should be detected through several methods, either luciferase reporter assays or high-throughput sequencing-based assays, such as *cis*-regulatory element analysis by sequencing (CRE-seq) (Kwasnieski et al., 2014) and self-transcribing active regulatory region sequencing (STARR-seq) (Arnold et al., 2013). Furthermore, functional verification is required to determine the direct biological significances, such as the oncogenic properties, which can be manifested though cancer cellular experiments (e.g., cell proliferation, cell cycle, cell death, migration, and invasion tests) along with *in vivo* model assays. In addition, other approaches are needed to be explored to demonstrate the effects of genetic variants within introns, exons, or intergenic regions. For instance, genetic variants mapping to exons of a lincRNA may alter the lincRNA secondary structure, which can be partly predicted using RNAfold web server (Hofacker and Stadler, 2006). The 5′ UTR (un-translated region) variants may affect the process of splicing and stability of RNA conformation, a functional splicing reporter minigene assay should be used to assess the effect of genetic variants on RNA splicing (Giorgi et al., 2015). Through the aforementioned knowable strategies, comprehensive functional verification of non-coding variants is very important to understand their biological consequence; there is an urgent need to explore more practical methods and strategies for functional verification research.

## Perspectives and Discussion

Single-nucleotide variants are the most common genetic variants and universally present in the human genome, including non-coding regions. One current belief is that heterogeneous disease (e.g., cancers susceptibility) may be caused by the accumulation of multiple driving genetic variations. GWASs have identified a large number of disease or trait-associated SNVs, and many of those are located in non-coding regions of the human genome. The functions of genetic variants are generally unknown and remain to be elucidated. One critical common viewpoint is that the significance of lincRNA variants depends on their genomic position. Certain SNVs are located in regulatory regions of lincRNA genes; it is found to affect the binding efficiency of transcription factor; it is known to possibly regulate the transcription of lincRNAs and other related genes in a cell-type- or tissue-specific manner. The mapping of SNVs to lincRNA transcript itself potentially affects the process of splicing and stability of lincRNA conformation or modulates the lincRNA secondary structure; these effects may lead to an alteration of the interactive properties and regulatory functions of lincRNA (Khurana et al., 2016). Collectively, these findings indicate that genetic variants in lincRNA regions may serve as a regulatory signature for early events, which illustrate the genomic background of lincRNA differential expressions in a tissue- or disorder-specific manner.

Considering their important regulatory role, lincRNAs may serve as the promising biomarkers for the diagnosis, prognosis, and treatment response of various diseases (Zou et al., 2018). With their characteristic of tissue and disease specificity, lincRNAs may be explored as target molecules for personalized medicine in the future (Huarte, 2015). Currently, molecular targets drug approved by the US Food and Drug Administration (FDA) are mainly derived from proteins. However, owing to the finiteness of druggable protein genes in the human genome, the expansion of potentially druggable targets may need to include lncRNA molecules. One lincRNA *PCA3*-based test (the PROGENSA *PCA3* assay approved by the FDA) has been used as a marker for the detection of prostate cancers (Evaluation of Genomic Applications in Practice and Prevention [EGAPP] Working Group, 2014). Moreover, a novel treatment strategy differs from the classical small molecules and antibodies that mainly target proteins. RNA-targeting therapeutics refer to the use of oligonucleotides to target primarily RNA involved in various diseases for therapeutic efforts. Two major approaches are employed to target RNA: double-stranded RNA-mediated interference (RNAi) and antisense oligonucleotides (ASOs) (Kole et al., 2012). Currently, both methods are in clinical trials. Among them, nusinersen (Spinraza), an ASO-targeting drug for spinal muscular atrophy (SMA), has been approved by the FDA (Finkel et al., 2017); patisiran, an RNAi therapeutic strategy for hereditary transthyretin amyloidosis (hATTR), has also shown promising results (Adams et al., 2018). Hence, we can expect that lincRNA molecules will provide additional options for RNA therapeutics. Importantly, disease-associated variants are found to exhibit a higher frequency in non-coding regions, which encompass enhancers, promoters, and other regulatory elements. It is likely that the role of genetic variants in lincRNA regions should be characterized at the regulatory network level. Genetic variants may offer the possibility to make use of the information from adjacent protein-coding or non-coding regions to link with heterogeneous diseases. Therefore, a combination of SNVs, lincRNAs, and proteins may bring personalized medicine closer to clinical applications in the foreseeable future (Li et al., 2019).

Previous studies may appear to be a slightly biased against the genetic variants that are located in non-coding regions, as their significant roles have not yet been explored to the same extent as those of protein-coding genes. In particular, for the disease-associated variants in lincRNA regions, whether functionally affected or altered in lincRNA expression by risk variants, it may be responsible for the disease development and its pathogenesis. Verification of the mechanisms requires a detailed understanding of the lincRNA structure and function, and a suitable experimental system to distinguish the subtle differences caused by genetic variants. Although it is difficult to describe the consequences of genetic variants in non-coding regions, more emerging technologies and approaches are urgently needed to explore the driving effects of genetic variants on lincRNA regions.

## Author Contributions

HZ consulted relevant literatures, finished the manuscript, and completed English revision. LT completed the figures and tables. F-FS provided constructive feedback and guidance. L-XW and H-HZ completed critical revisions and proofread the manuscript.

## Conflict of Interest

The authors declare that the research was conducted in the absence of any commercial or financial relationships that could be construed as a potential conflict of interest.

## Footnotes

1 http://genome.ucsc.edu/ENCODE/

## References

[B1] Adams D. Gonzalez-Duarte A. O’riordan W. D. Yang C. C. Ueda M. Kristen A. V. (2018). Patisiran, an RNAi therapeutic, for hereditary transthyretin amyloidosis. *N. Engl. J. Med.* 379 11–21. 10.1056/NEJMoa1716153 29972753

[B2] Arnold C. D. Gerlach D. Stelzer C. Boryn L. M. Rath M. Stark A. (2013). Genome-wide quantitative enhancer activity maps identified by STARR-seq. *Science* 339 1074–1077. 10.1126/science.1232542 23328393

[B3] Athanasiu L. Smorr L. L. Tesli M. Rossberg J. I. Sonderby I. E. Spigset O. (2015). Genome-wide association study identifies common variants associated with pharmacokinetics of psychotropic drugs. *J. Psychopharmacol.* 29 884–891. 10.1177/0269881115584469 25944848

[B4] Cabili M. N. Trapnell C. Goff L. Koziol M. Tazon-Vega B. Regev A. (2011). Integrative annotation of human large intergenic noncoding RNAs reveals global properties and specific subclasses. *Genes Dev.* 25 1915–1927. 10.1101/gad.17446611 21890647 PMC3185964

[B5] Cerhan J. R. Berndt S. I. Vijai J. Ghesquieres H. Mckay J. Wang S. S. (2014). Genome-wide association study identifies multiple susceptibility loci for diffuse large B cell lymphoma. *Nat. Genet.* 46 1233–1238. 10.1038/ng.3105 25261932 PMC4213349

[B6] Chen X. Hao Y. Cui Y. Fan Z. He S. Luo J. (2017). LncVar: a database of genetic variation associated with long non-coding genes. *Bioinformatics* 33 112–118. 10.1093/bioinformatics/btw581 27605101

[B7] Chen X. F. Zhu D. L. Yang M. Hu W. X. Duan Y. Y. Lu B. J. (2018). An Osteoporosis Risk SNP at 1p36.*12* Acts as an Allele-Specific Enhancer to Modulate LINC00339 Expression via Long-Range Loop Formation. *Am. J. Hum. Genet.* 102 776–793. 10.1016/j.ajhg.2018.03.001 29706346 PMC5986728

[B8] Chu H. Xia L. Qiu X. Gu D. Zhu L. Jin J. (2015). Genetic variants in noncoding PIWI-interacting RNA and colorectal cancer risk. *Cancer* 121 2044–2052. 10.1002/cncr.29314 25740697

[B9] Chu M. Yuan W. Wu S. Wang Z. Mao L. Tian T. (2016). Quantitative assessment of polymorphisms in H19 lncRNA and cancer risk: a meta-analysis of 13,392 cases and 18,893 controls. *Oncotarget* 7 78631–78639. 10.18632/oncotarget.12530 27732938 PMC5346665

[B10] Chung S. Nakagawa H. Uemura M. Piao L. Ashikawa K. Hosono N. (2011). Association of a novel long non-coding RNA in 8q24 with prostate cancer susceptibility. *Cancer Sci.* 102 245–252. 10.1111/j.1349-7006.2010.01737.x 20874843

[B11] Conti D. V. Wang K. Sheng X. Bensen J. T. Hazelett D. J. Cook M. B. (2017). Two novel susceptibility loci for prostate cancer in men of african ancestry. *J. Natl. Cancer Inst.* 109:djx084 10.1093/jnci/djx084 PMC5448553 29117387

[B12] Cui Z. Gao M. Yin Z. Yan L. Cui L. (2018). Association between lncRNA CASC8 polymorphisms and the risk of cancer: a meta-analysis. *Cancer Manag. Res.* 10 3141–3148. 10.2147/CMAR.S170783 30214306 PMC6124472

[B13] Derrien T. Johnson R. Bussotti G. Tanzer A. Djebali S. Tilgner H. (2012). The GENCODE v7 catalog of human long noncoding RNAs: analysis of their gene structure, evolution, and expression. *Genome Res.* 22 1775–1789. 10.1101/gr.132159.111 22955988 PMC3431493

[B14] Evaluation of Genomic Applications in Practice and Prevention [EGAPP] Working Group (2014). Recommendations from the EGAPP Working Group: does PCA3 testing for the diagnosis and management of prostate cancer improve patient health outcomes?. *Genet. Med.* 16 338–346. 10.1038/gim.2013.141 24071797

[B15] Fava V. M. Manry J. Cobat A. Orlova M. Van Thuc N. Moraes M. O. (2017). A genome wide association study identifies a lncRna as risk factor for pathological inflammatory responses in leprosy. *PLoS Genet.* 13:e1006637. 10.1371/journal.pgen.1006637 28222097 PMC5340414

[B16] Finkel R. S. Mercuri E. Darras B. T. Connolly A. M. Kuntz N. L. Kirschner J. (2017). Nusinersen versus sham control in infantile-onset spinal muscular atrophy. *N. Engl. J. Med.* 377 1723–1732. 10.1056/NEJMoa1702752 29091570

[B17] Freedman M. L. Monteiro A. N. Gayther S. A. Coetzee G. A. Risch A. Plass C. (2011). Principles for the post-GWAS functional characterization of cancer risk loci. *Nat. Genet.* 43 513–518. 10.1038/ng.840 21614091 PMC3325768

[B18] Gao P. Xia J. H. Sipeky C. Dong X. M. Zhang Q. Yang Y. (2018). Biology AND CLINICAL IMPLICATIONS OF the 19q13 aggressive prostate cancer susceptibility locus. *Cell* 174 576.e18–589.e18. 10.1016/j.cell.2018.06.003 30033361 PMC6091222

[B19] Gao W. Zhu M. Wang H. Zhao S. Zhao D. Yang Y. (2015). Association of polymorphisms in long non-coding RNA H19 with coronary artery disease risk in a Chinese population. *Mutat. Res.* 772 15–22. 10.1016/j.mrfmmm.2014.12.009 25772106

[B20] Ge L. Wang Q. Hu S. Yang X. (2018). Rs217727 polymorphism in H19 promotes cell apoptosis by regulating the expressions of H19 and the activation of its downstream signaling pathway. *J. Cell Physiol.* 234 7279–7291. 10.1002/jcp.27485 30362559

[B21] Genomes Project C. Auton A. Brooks L. D. Durbin R. M. Garrison E. P. Kang H. M. (2015). A global reference for human genetic variation. *Nature* 526 68–74. 10.1038/nature15393 26432245 PMC4750478

[B22] Ghanbari M. Peters M. J. De Vries P. S. Boer C. G. Van Rooij J. G. J. Lee Y. C. (2018). A systematic analysis highlights multiple long non-coding RNAs associated with cardiometabolic disorders. *J. Hum. Genet.* 63 431–446. 10.1038/s10038-017-0403-x 29382920 PMC6894420

[B23] Ghesquieres H. Larrabee B. R. Casasnovas O. Maurer M. J. Mckay J. D. Ansell S. M. (2018). A susceptibility locus for classical Hodgkin lymphoma at 8q24 near MYC/PVT1 predicts patient outcome in two independent cohorts. *Br. J. Haematol.* 180 286–290. 10.1111/bjh.14306 27716907 PMC5344766

[B24] Giorgi G. Casarin A. Trevisson E. Dona M. Cassina M. Graziano C. (2015). Validation of CFTR intronic variants identified during cystic fibrosis population screening by a minigene splicing assay. *Clin. Chem. Lab. Med* 53 1719–1723. 10.1515/cclm-2014-1047 25781545

[B25] Gong W. J. Peng J. B. Yin J. Y. Li X. P. Zheng W. Xiao L. (2017). Association between well-characterized lung cancer lncRNA polymorphisms and platinum-based chemotherapy toxicity in Chinese patients with lung cancer. *Acta Pharmacol. Sin.* 38 581–590. 10.1038/aps.2016.164 28260796 PMC5386317

[B26] GTEx Consortium, Data Analysis &Coordinating Center [LDACC], Analysis Working Group, Statistical Methods groups, Analysis Working Group, Enhancing GTEx groups (2017). Genetic effects on gene expression across human tissues. *Nature* 550 204–213. 10.1038/nature25160 29022597 PMC5776756

[B27] Guo H. Ahmed M. Zhang F. Yao C. Q. Li S. Liang Y. (2016). Modulation of long noncoding RNAs by risk SNPs underlying genetic predispositions to prostate cancer. *Nat. Genet.* 48 1142–1150. 10.1038/ng.3637 27526323

[B28] Guo Q. Y. Wang H. Wang Y. (2017). LncRNA H19 polymorphisms associated with the risk of OSCC in Chinese population. *Eur. Rev. Med. Pharmacol. Sci.* 21 3770–3774. 28975993

[B29] Guo Z. Wang Y. Zhao Y. Jin Y. An L. Wu B. (2017). Genetic polymorphisms of long non-coding RNA GAS5 predict platinum-based concurrent chemoradiotherapy response in nasopharyngeal carcinoma patients. *Oncotarget* 8 62286–62297. 10.18632/oncotarget.19725 28977945 PMC5617505

[B30] Han L. Liu S. Liang J. Guo Y. Shen S. Guo X. (2017). A genetic polymorphism at miR-526b binding-site in the lincRNA-NR_024015 exon confers risk of esophageal squamous cell carcinoma in a population of North China. *Mol. Carcinog.* 56 960–971. 10.1002/mc.22549 27583835

[B31] Hanson R. L. Craig D. W. Millis M. P. Yeatts K. A. Kobes S. Pearson J. V. (2007). Identification of PVT1 as a candidate gene for end-stage renal disease in type 2 diabetes using a pooling-based genome-wide single nucleotide polymorphism association study. *Diabetes Metab. Res. Rev.* 56 975–983. 10.2337/db06-1072 17395743

[B32] Harati-Sadegh M. Kohan L. Teimoori B. Salimi S. (2018). The long non-coding RNA H19 rs217727 polymorphism is associated with PE susceptibility. *J. Cell. Biochem.* 119 5473–5480. 10.1002/jcb.26708 29380421

[B33] He H. Li W. Liyanarachchi S. Srinivas M. Wang Y. Akagi K. (2015). Multiple functional variants in long-range enhancer elements contribute to the risk of SNP rs965513 in thyroid cancer. *Proc. Natl. Acad. Sci. U.S.A.* 112 6128–6133. 10.1073/pnas.1506255112 25918370 PMC4434723

[B34] Hindorff L. A. Sethupathy P. Junkins H. A. Ramos E. M. Mehta J. P. Collins F. S. (2009). Potential etiologic and functional implications of genome-wide association loci for human diseases and traits. *Proc. Natl. Acad. Sci. U.S.A.* 106 9362–9367. 10.1073/pnas.0903103106 19474294 PMC2687147

[B35] Hofacker I. L. Stadler P. F. (2006). Memory efficient folding algorithms for circular RNA secondary structures. *Bioinformatics* 22 1172–1176. 10.1093/bioinformatics/btl023 16452114

[B36] Hon C. C. Ramilowski J. A. Harshbarger J. Bertin N. Rackham O. J. Gough J. (2017). An atlas of human long non-coding RNAs with accurate 5’ ends. *Nature* 543 199–204. 10.1038/nature21374 28241135 PMC6857182

[B37] Hu L. Chen S. H. Lv Q. L. Sun B. Qu Q. Qin C. Z. (2016). Clinical Significance of Long Non-Coding RNA CASC8 rs10505477 polymorphism in lung cancer susceptibility, platinum-based chemotherapy response, and toxicity. *Int. J. Environ. Res. Public Health* 13:545 10.3390/ijerph13060545 PMC4924002 27249003

[B38] Hua J. T. Ahmed M. Guo H. Zhang Y. Chen S. Soares F. (2018). Risk SNP-mediated promoter-enhancer switching drives prostate cancer through lncRNA PCAT19. *Cell* 174 564.e18–575.e18. 10.1016/j.cell.2018.06.014 30033362

[B39] Huang D. Yi X. Zhang S. Zheng Z. Wang P. Xuan C. (2018). GWAS4D: multidimensional analysis of context-specific regulatory variant for human complex diseases and traits. *Nucleic Acids Res.* 46 W114–W120. 10.1093/nar/gky407 29771388 PMC6030885

[B40] Huarte M. (2015). The emerging role of lncRNAs in cancer. *Nat. Med.* 21 1253–1261. 10.1038/nm.3981 26540387

[B41] Human Genome Structural Variation Working Group, Eichler E. E. Nickerson D. A. Altshuler D. Bowcock A. M. Brooks L. D. (2007). Completing the map of human genetic variation. *Nature* 447 161–165. 10.1038/447161a 17495918 PMC2685471

[B42] Ingle J. N. Xie F. Ellis M. J. Goss P. E. Shepherd L. E. Chapman J. W. (2016). Genetic polymorphisms in the long noncoding RNA MIR2052HG offer a pharmacogenomic basis for the response of breast cancer patients to aromatase inhibitor therapy. *Cancer Res.* 76 7012–7023. 10.1158/0008-5472.CAN-16-1371 27758888 PMC5135610

[B43] Ishii N. Ozaki K. Sato H. Mizuno H. Saito S. Takahashi A. (2006). Identification of a novel non-coding RNA, MIAT, that confers risk of myocardial infarction. *J. Hum. Genet.* 51 1087–1099. 10.1007/s10038-006-0070-9 17066261

[B44] Jendrzejewski J. He H. Radomska H. S. Li W. Tomsic J. Liyanarachchi S. (2012). The polymorphism rs944289 predisposes to papillary thyroid carcinoma through a large intergenic noncoding RNA gene of tumor suppressor type. *Proc. Natl. Acad. Sci. U.S.A.* 109 8646–8651. 10.1073/pnas.1205654109 22586128 PMC3365219

[B45] Kenyan Bacteraemia Study Group, Wellcome Trust , Case Control C. Rautanen A. Pirinen M. Mills T. C. (2016). Polymorphism in a lincRNA associates with a doubled risk of pneumococcal bacteremia in kenyan children. *Am. J. Hum. Genet.* 98 1092–1100. 10.1016/j.ajhg.2016.03.025 27236921 PMC4908194

[B46] Khurana E. Fu Y. Chakravarty D. Demichelis F. Rubin M. A. Gerstein M. (2016). Role of non-coding sequence variants in cancer. *Nat. Rev. Genet.* 17 93–108. 10.1038/nrg.2015.17 26781813

[B47] Kim T. Cui R. Jeon Y. J. Lee J. H. Lee J. H. Sim H. (2014). Long-range interaction and correlation between MYC enhancer and oncogenic long noncoding RNA CARLo-5. *Proc. Natl. Acad. Sci. U.S.A.* 111 4173–4178. 10.1073/pnas.1400350111 24594601 PMC3964128

[B48] Kole R. Krainer A. R. Altman S. (2012). RNA therapeutics: beyond RNA interference and antisense oligonucleotides. *Nat. Rev. Drug Discov.* 11 125–140. 10.1038/nrd3625 22262036 PMC4743652

[B49] Konermann S. Brigham M. D. Trevino A. E. Joung J. Abudayyeh O. O. Barcena C. (2015). Genome-scale transcriptional activation by an engineered CRISPR-Cas9 complex. *Nature* 517 583–588. 10.1038/nature14136 25494202 PMC4420636

[B50] Kretz M. Siprashvili Z. Chu C. Webster D. E. Zehnder A. Qu K. (2013). Control of somatic tissue differentiation by the long non-coding RNA TINCR. *Nature* 493 231–235. 10.1038/nature11661 23201690 PMC3674581

[B51] Kulakova O. Bashinskaya V. Kiselev I. Baulina N. Tsareva E. Nikolaev R. (2017). Pharmacogenetics of glatiramer acetate therapy for multiple sclerosis: the impact of genome-wide association studies identified disease risk loci. *Pharmacogenomics* 18 1563–1574. 10.2217/pgs-2017-0058 29095108

[B52] Kwasnieski J. C. Fiore C. Chaudhari H. G. Cohen B. A. (2014). High-throughput functional testing of ENCODE segmentation predictions. *Genome Res.* 24 1595–1602. 10.1101/gr.173518.114 25035418 PMC4199366

[B53] Lee T. H. Ko T. M. Chen C. H. Lee M. T. Chang Y. J. Chang C. H. (2016). Identification of PTCSC3 as a novel locus for large-vessel ischemic stroke: a genome-wide association Study. *J. Am. Heart Assoc.* 5:e003003. 10.1161/JAHA.115.003003 27025970 PMC4943273

[B54] Li C. Rao T. Chen X. Zou Z. Wei A. Tang J. (2019). HLA-B^∗^35:01 allele is a potential biomarker for predicting polygonum multiflorum-induced liver injury in humans. *Hepatology* 70 346–357. 10.1002/hep.30660 30985007

[B55] Li L. Sun R. Liang Y. Pan X. Li Z. Bai P. (2013). Association between polymorphisms in long non-coding RNA PRNCR1 in 8q24 and risk of colorectal cancer. *J. Exp. Clin. Cancer Res.* 32:104. 10.1186/1756-9966-32-104 24330491 PMC4029281

[B56] Li Q. Ma G. Sun S. Xu Y. Wang B. (2018a). Polymorphism in the promoter region of lncRNA GAS5 is functionally associated with the risk of gastric cancer. *Clin. Res. Hepatol. Gastroenterol.* 42 478–482. 10.1016/j.clinre.2018.01.006 29602737

[B57] Li Q. Zhu W. Zhang B. Wu Y. Yan S. Yuan Y. (2018b). The MALAT1 gene polymorphism and its relationship with the onset of congenital heart disease in Chinese. *Biosci. Rep.* 38:BSR20171381 10.1042/BSR20171381 PMC6048208 29559566

[B58] Li Y. Bao C. Gu S. Ye D. Jing F. Fan C. (2017). Associations between novel genetic variants in the promoter region of MALAT1 and risk of colorectal cancer. *Oncotarget* 8 92604–92614. 10.18632/oncotarget.21507 29190941 PMC5696207

[B59] Ling H. Spizzo R. Atlasi Y. Nicoloso M. Shimizu M. Redis R. S. (2013). CCAT2, a novel noncoding RNA mapping to 8q24, underlies metastatic progression and chromosomal instability in colon cancer. *Genome Res.* 23 1446–1461. 10.1101/gr.152942.112 23796952 PMC3759721

[B60] Ma G. Gu D. Lv C. Chu H. Xu Z. Tong N. (2015). Genetic variant in 8q24 is associated with prognosis for gastric cancer in a Chinese population. *J. Gastroenterol. Hepatol.* 30 689–695. 10.1111/jgh.12801 25302443

[B61] MacArthur J. Bowler E. Cerezo M. Gil L. Hall P. Hastings E. (2017). The new NHGRI-EBI Catalog of published genome-wide association studies (GWAS Catalog). *Nucleic Acids Res.* 45 D896–D901. 10.1093/nar/gkw1133 27899670 PMC5210590

[B62] Maris J. M. Mosse Y. P. Bradfield J. P. Hou C. Monni S. Scott R. H. (2008). Chromosome 6p22 locus associated with clinically aggressive neuroblastoma. *N. Engl. J. Med.* 358 2585–2593. 10.1056/NEJMoa0708698 18463370 PMC2742373

[B63] Marjonen H. Auvinen P. Kahila H. Tsuiko O. Koks S. Tiirats A. (2018). rs10732516 polymorphism at the IGF2/H19 locus associates with genotype-specific effects on placental DNA methylation and birth weight of newborns conceived by assisted reproductive technology. *Clin. Epigenetics* 10:80. 10.1186/s13148-018-0511-2 29946374 PMC6006593

[B64] Meyer K. B. Maia A. T. O’reilly M. Ghoussaini M. Prathalingam R. Porter-Gill P. (2011). A functional variant at a prostate cancer predisposition locus at 8q24 is associated with PVT1 expression. *PLoS Genet.* 7:e1002165. 10.1371/journal.pgen.1002165 21814516 PMC3140991

[B65] Miao Y. R. Liu W. Zhang Q. Guo A. Y. (2018). lncRNASNP2: an updated database of functional SNPs and mutations in human and mouse lncRNAs. *Nucleic Acids Res.* 46 D276–D280. 10.1093/nar/gkx1004 29077939 PMC5753387

[B66] Ng M. C. Y. Graff M. Lu Y. Justice A. E. Mudgal P. Liu C. T. (2017). Discovery and fine-mapping of adiposity loci using high density imputation of genome-wide association studies in individuals of African ancestry: African ancestry anthropometry genetics Consortium. *PLoS Genet.* 13:e1006719. 10.1371/journal.pgen.1006719 28430825 PMC5419579

[B67] Nikpay M. Goel A. Won H. H. Hall L. M. Willenborg C. Kanoni S. (2015). A comprehensive 1,000 Genomes-based genome-wide association meta-analysis of coronary artery disease. *Nat. Genet.* 47 1121–1130. 10.1038/ng.3396 26343387 PMC4589895

[B68] Ning S. Yue M. Wang P. Liu Y. Zhi H. Zhang Y. (2017). LincSNP 2.0: an updated database for linking disease-associated SNPs to human long non-coding RNAs and their TFBSs. *Nucleic Acids Res.* 45 D74–D78. 10.1093/nar/gkw945 27924020 PMC5210641

[B69] Orr N. Cooke R. Jones M. Fletcher O. Dudbridge F. Chilcott-Burns S. (2011). Genetic variants at chromosomes 2q35, 5p12, 6q25.1, 10q26.13, and 16q12.1 influence the risk of breast cancer in men. *PLoS Genet.* 7:e1002290. 10.1371/journal.pgen.1002290 21949660 PMC3174231

[B70] Petry C. J. Seear R. V. Wingate D. L. Acerini C. L. Ong K. K. Hughes I. A. (2011). Maternally transmitted foetal H19 variants and associations with birth weight. *Hum. Genet.* 130 663–670. 10.1007/s00439-011-1005-x 21573965

[B71] Pomerantz M. M. Ahmadiyeh N. Jia L. Herman P. Verzi M. P. Doddapaneni H. (2009). The 8q24 cancer risk variant rs6983267 shows long-range interaction with MYC in colorectal cancer. *Nat. Genet.* 41 882–884. 10.1038/ng.403 19561607 PMC2763485

[B72] Ponting C. P. Oliver P. L. Reik W. (2009). Evolution and functions of long noncoding RNAs. *Cell* 136 629–641. 10.1016/j.cell.2009.02.006 19239885

[B73] Powell J. E. Fung J. N. Shakhbazov K. Sapkota Y. Cloonan N. Hemani G. (2016). Endometriosis risk alleles at 1p36.12 act through inverse regulation of CDC42 and LINC00339. *Hum. Mol. Genet.* 25 5046–5058. 10.1093/hmg/ddw320 28171565

[B74] Rao S. Q. Hu H. L. Ye N. Shen Y. Xu Q. (2015). Genetic variants in long non-coding RNA MIAT contribute to risk of paranoid schizophrenia in a Chinese Han population. *Schizophr. Res.* 166 125–130. 10.1016/j.schres.2015.04.032 26004688

[B75] Redis R. S. Vela L. E. Lu W. Ferreira De Oliveira J. Ivan C. Rodriguez-Aguayo C. (2016). Allele-specific reprogramming of cancer metabolism by the long non-coding RNA CCAT2. *Mol. Cell.* 61:640 10.1016/j.molcel.2016.02.006 28934601

[B76] Schaub M. A. Boyle A. P. Kundaje A. Batzoglou S. Snyder M. (2012). Linking disease associations with regulatory information in the human genome. *Genome Res.* 22 1748–1759. 10.1101/gr.136127.111 22955986 PMC3431491

[B77] Shah M. Y. Ferracin M. Pileczki V. Chen B. Redis R. Fabris L. (2018). Cancer-associated rs6983267 SNP and its accompanying long noncoding RNA CCAT2 induce myeloid malignancies via unique SNP-specific RNA mutations. *Genome Res.* 28 432–447. 10.1101/gr.225128.117 29567676 PMC5880235

[B78] Shyn S. I. Shi J. Kraft J. B. Potash J. B. Knowles J. A. Weissman M. M. (2011). Novel loci for major depression identified by genome-wide association study of sequenced treatment alternatives to relieve depression and meta-analysis of three studies. *Mol. Psychiatry* 16 202–215. 10.1038/mp.2009.125 20038947 PMC2888856

[B79] Sur I. K. Hallikas O. Vaharautio A. Yan J. Turunen M. Enge M. (2012). Mice lacking a Myc enhancer that includes human SNP rs6983267 are resistant to intestinal tumors. *Science* 338 1360–1363. 10.1126/science.1228606 23118011

[B80] Tao R. Hu S. Wang S. Zhou X. Zhang Q. Wang C. (2015). Association between indel polymorphism in the promoter region of lncRNA GAS5 and the risk of hepatocellular carcinoma. *Carcinogenesis* 36 1136–1143. 10.1093/carcin/bgv099 26163879

[B81] Teerlink C. C. Leongamornlert D. Dadaev T. Thomas A. Farnham J. Stephenson R. A. (2016). Genome-wide association of familial prostate cancer cases identifies evidence for a rare segregating haplotype at 8q24.21. *Hum. Genet.* 135 923–938. 10.1007/s00439-016-1690-6 27262462 PMC5020907

[B82] The Encode Project Consortium (2012). An integrated encyclopedia of DNA elements in the human genome. *Nature* 489 57–74. 10.1038/nature11247 22955616 PMC3439153

[B83] Tuupanen S. Turunen M. Lehtonen R. Hallikas O. Vanharanta S. Kivioja T. (2009). The common colorectal cancer predisposition SNP rs6983267 at chromosome 8q24 confers potential to enhanced Wnt signaling. *Nat. Genet.* 41 885–890. 10.1038/ng.406 19561604

[B84] Ulitsky I. Bartel D. P. (2013). lincRNAs: genomics, evolution, and mechanisms. *Cell* 154 26–46. 23827673 10.1016/j.cell.2013.06.020 PMC3924787

[B85] Venter J. C. Adams M. D. Myers E. W. Li P. W. Mural R. J. Sutton G. G. (2001). The sequence of the human genome. *Science* 291 1304–1351. 11181995 10.1126/science.1058040

[B86] Verhaegh G. W. Verkleij L. Vermeulen S. H. Den Heijer M. Witjes J. A. Kiemeney L. A. (2008). Polymorphisms in the H19 gene and the risk of bladder cancer. *Eur. Urol.* 54 1118–1126. 10.1016/j.eururo.2008.01.060 18262338

[B87] Wallace C. Smyth D. J. Maisuria-Armer M. Walker N. M. Todd J. A. Clayton D. G. (2010). The imprinted DLK1-MEG3 gene region on chromosome 14q32.2 alters susceptibility to type 1 diabetes. *Nat. Genet.* 42 68–71. 10.1038/ng.493 19966805 PMC2820243

[B88] Wang B. G. Lv Z. Ding H. X. Fang X. X. Wen J. Xu Q. (2018a). The association of lncRNA-HULC polymorphisms with hepatocellular cancer risk and prognosis. *Gene* 670 148–154. 10.1016/j.gene.2018.05.096 29803923

[B89] Wang B. G. Xu Q. Lv Z. Fang X. X. Ding H. X. Wen J. (2018b). Association of twelve polymorphisms in three onco-lncRNA genes with hepatocellular cancer risk and prognosis: a case-control study. *World J. Gastroenterol.* 24 2482–2490. 10.3748/wjg.v24.i23.2482 29930469 PMC6010940

[B90] Wang J. Z. Xiang J. J. Wu L. G. Bai Y. S. Chen Z. W. Yin X. Q. (2017). A genetic variant in long non-coding RNA MALAT1 associated with survival outcome among patients with advanced lung adenocarcinoma: a survival cohort analysis. *BMC Cancer* 17:167. 10.1186/s12885-017-3151-6 28253859 PMC5335789

[B91] Westra H. J. Martinez-Bonet M. Onengut-Gumuscu S. Lee A. Luo Y. Teslovich N. (2018). Fine-mapping and functional studies highlight potential causal variants for rheumatoid arthritis and type 1 diabetes. *Nat. Genet.* 50 1366–1374. 10.1038/s41588-018-0216-7 30224649 PMC6364548

[B92] Wu H. Zheng J. Deng J. Hu M. You Y. Li N. (2013). A genetic polymorphism in lincRNA-uc003opf.1 is associated with susceptibility to esophageal squamous cell carcinoma in Chinese populations. *Carcinogenesis* 34 2908–2917. 10.1093/carcin/bgt252 23872665

[B93] Wu N. Y. Huang H. S. Chao T. H. Chou H. M. Fang C. Qin C. Z. (2017). Progesterone prevents high-grade serous ovarian cancer by inducing necroptosis of p53-defective fallopian tube epithelial cells. *Cell Rep.* 18 2557–2565. 10.1016/j.celrep.2017.02.049 28297660

[B94] Wu Q. Yan W. Han R. Yang J. Yuan J. Ji X. (2016). Polymorphisms in long noncoding RNA H19 contribute to the protective effects of coal workers’ pneumoconiosis in a chinese population. *Int. J. Environ. Res. Public Health* 13:903 10.3390/ijerph13090903 PMC5036736 27626436

[B95] Xue Y. Gu D. Ma G. Zhu L. Hua Q. Chu H. (2015). Genetic variants in lncRNA HOTAIR are associated with risk of colorectal cancer. *Mutagenesis* 30 303–310. 10.1093/mutage/geu076 25432874

[B96] Xue Y. Wang M. Kang M. Wang Q. Wu B. Chu H. (2013). Association between lncrna PCGEM1 polymorphisms and prostate cancer risk. *Prostate Cancer Prostatic Dis.* 16 139–144. 10.1038/pcan.2013.6 23459097

[B97] Yan H. Zhang D. Y. Li X. Yuan X. Q. Yang Y. L. Zhu K. W. (2017). Long non-coding RNA GAS5 polymorphism predicts a poor prognosis of acute myeloid leukemia in Chinese patients via affecting hematopoietic reconstitution. *Leuk. Lymphoma* 58 1948–1957. 10.1080/10428194.2016.1266626 27951730

[B98] Yang M. L. Huang Z. Wang Q. Chen H. H. Ma S. N. Wu R. (2018). The association of polymorphisms in lncRNA-H19 with hepatocellular cancer risk and prognosis. *Biosci. Rep.* 38:BSR20171652 10.1042/BSR20171652 PMC6123070 29511035

[B99] Yeager M. Orr N. Hayes R. B. Jacobs K. B. Kraft P. Wacholder S. (2007). Genome-wide association study of prostate cancer identifies a second risk locus at 8q24. *Nat. Genet.* 39 645–649. 10.1016/j.eururo.2007.06.013 17401363

[B100] Zhang Y. Manjunath M. Zhang S. Chasman D. Roy S. Song J. S. (2018). Integrative genomic analysis predicts causative Cis-regulatory mechanisms of the breast cancer-associated genetic variant rs4415084. *Cancer Res.* 78 1579–1591. 10.1158/0008-5472.CAN-17-3486 29351903 PMC5882544

[B101] Zhang Z. Zhu Z. Zhang B. Li W. Li X. Wu X. (2014). Frequent mutation of rs13281615 and its association with PVT1 expression and cell proliferation in breast cancer. *J. Genet. Genomics* 41 187–195. 10.1016/j.jgg.2014.03.006 24780616

[B102] Zhao X. Wei X. Zhao L. Shi L. Cheng J. Kang S. (2016). The rs6983267 SNP and long non-coding RNA CARLo-5 are associated with endometrial carcinoma. *Environ. Mol. Mutagen.* 57 508–515. 10.1002/em.22031 27432114

[B103] Zheng J. Huang X. Tan W. Yu D. Du Z. Chang J. (2016). Pancreatic cancer risk variant in LINC00673 creates a miR-1231 binding site and interferes with PTPN11 degradation. *Nat. Genet.* 48 747–757. 10.1038/ng.3568 27213290

[B104] Zheng Y. Yang C. Tong S. Ding Y. Deng W. Song D. (2017). Genetic variation of long non-coding RNA TINCR contribute to the susceptibility and progression of colorectal cancer. *Oncotarget* 8 33536–33543. 10.18632/oncotarget.16538 28418933 PMC5464888

[B105] Zhuo Y. Zeng Q. Zhang P. Li G. Xie Q. Cheng Y. (2017). Functional polymorphism of lncRNA MALAT1 contributes to pulmonary arterial hypertension susceptibility in Chinese people. *Clin. Chem. Lab. Med* 55 38–46. 10.1515/cclm-2016-0056 27362960

[B106] Zou H. Wen C. Peng Z. Shao Y. Hu L. Li S. (2018). P4HB and PDIA3 are associated with tumor progression and therapeutic outcome of diffuse gliomas. *Oncol. Rep.* 39 501–510. 10.3892/or.2017.6134 29207176 PMC5783617

[B107] Zou H. Wu L. X. Yang Y. Li S. Mei Y. Liu Y. B. (2017). lncRNAs PVT1 and HAR1A are prognosis biomarkers and indicate therapy outcome for diffuse glioma patients. *Oncotarget* 8 78767–78780. 10.18632/oncotarget.20226 29108264 PMC5667997

